# Changing perceptions of general health in the Kayseri Province, Turkey in 2004 and 2017: A population-based study

**DOI:** 10.3389/fpubh.2023.1095163

**Published:** 2023-02-24

**Authors:** Vesile Senol, Ferhan Elmali, Fevziye Cetinkaya, Melis Nacar

**Affiliations:** ^1^School of Health Science, Kapadokya University, Nevşehir, Türkiye; ^2^Department of Biostatistics, Medical School, Izmir Katip Çelebi University, Izmir, Türkiye; ^3^Department of Public Health, School of Medicine, Erciyes University, Kayseri, Türkiye; ^4^Department of Medical Education, Medical School, Erciyes University, Kayseri, Türkiye

**Keywords:** self-rated health, Nottingham Health Profile, quality of life, changing, overtime

## Abstract

**Aim:**

Self-rated health (SRH) and health-related quality of life (HRQoL) have closely related outcomes in measuring general health status in community-based studies. The aim of this study is to determine changes in the self-perceived overall health of people and affected factors by comparing the findings of two studies conducted in the same research area.

**Methods:**

Both studies were conducted using the same measurement tools in households determined by random sampling techniques in the same research areas. The first and second studies were conducted with 1,304 and 1,533 people residing in 501 and 801 households in 2004 and 2017, respectively. The demographic data form, the Nottingham Health Profile (NHP), and a single-item SRH questionnaire were used for data collection.

**Results:**

The rate of good SRH increased from 56% to 70% while the average NHP score decreased from 30.87 to 20.34. The predictors of negative health perceptions were the presence of chronic diseases (OR 3.4–2.7-times higher), being female (OR.1.4–1.5 times higher), and the completion of primary education only (OR. 2.7–2.8 times higher) both 2004 and 2017. Living 500–1,000 m from the nearest healthcare facility was the main protective variable against poor SRH.

**Conclusions:**

Good SRH and HRQoL have increased significantly over time. Chronic diseases, education, and gender are the strongest predictors of poor SRH.

## Introduction

Self-rated health (SRH) and health-related quality of life (HRQoL) are two outcome measures that are used to evaluate people's perceptions of their health status in population-based studies. Both of these measures are self-reported, inexpensive, and easy to conduct. SRH (also known as self-assessed health or self-perceived health) is evaluated according to the answer to a single-item question: “In general, how would you rate your health: poor, fair, good, very good, or excellent?” (1, 2). According to the World Health Organization, the SRH question is a simpler (3), less expensive (4), more precise and objective (5), and culturally sensitive (6) outcome measure than the clinical assessment tools (7). Despite this, the single item about which SRH is concerned is sufficient to reveal people's health status, but it cannot provide more specific health status information.

Self-perceived overall health can also be measured using HRQoL, which is often used in community-based studies and defines the general health perceptions of the individual's or the group's subjective health status (or QoL) in physical, social, and emotional domains (8, 9). It is determined by many factors and can be arranged according to several dimensions. A parameter related to HRQoL is self-rated health (1, 10). Self-perceived health (SPH) is a powerful and independent predictor that is affected by general and disease-specific mortality and the incidence rate of chronic disease and includes many components related to public health. Studies in this area state that SPH can be related to behavioral, biological, psychological, and social dimensions, such as general and functional status, age, gender, marital status, education, household income, chronic diseases, lifestyle factors, culture, health beliefs, and healthcare service utilization (11–22).

Self Perceived Health, is a powerful predictor which reflects the rate of use of health-care, can vary depending on time, structural-financial reforms, and epidemiological transformation. As a matter of fact that, McCallum et al. (23), Waidmann et al. (24), and Leinonen et al. (25) suggested that SRH follows a change in health.

The aim of this study is to determine the change in the self-perceived overall health status of people and the affecting factors by comparing two different years, using the same research methods.

## Materials and methods

### Study design and settings

**Figure 1:**
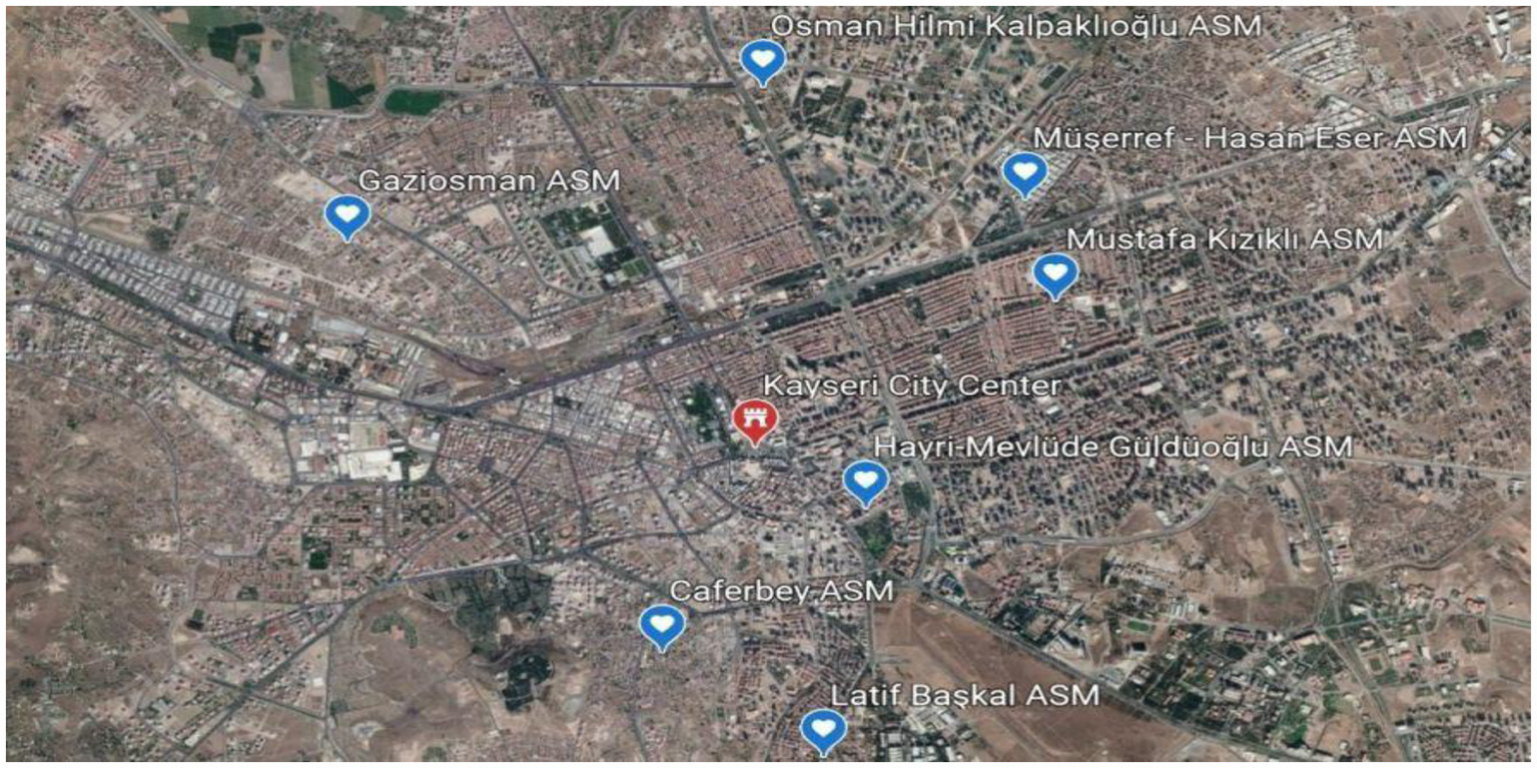
The map of research regions in the years 2004 and 2017.

This cross-sectional descriptive-analytic study is a two-part study, which was carried out in two different years, and describes the level of healthcare services used by people, their level of general health perception, and the change it has shown over time. The first of these studies was carried out in Kayseri in 2004 and the second one was carried out in 2017 in the same region (Figure 1). The findings of these studies on healthcare use, influencing factors, and changes in usage patterns will be published in a separate study due to an excess of data. This article contains the results regarding perceived health.

### Study population and sampling

This study was carried out in Kayseri, which is one of the biggest cities in Turkey and an important commercial and industrial center in central Anatolia. Its population is nearly 1.5 million.

In 2004, 21 urban Primary Health Centers (PHCs), and in 2017, 71 urban Family Health Centers (FHCs) provided healthcare services in the same region (Figure 1). With the Health Transformation Program in 2008, the healthcare service delivery model in Turkey was changed. In the provision of primary care services, the health center model was replaced by the family medicine system.

The study area was stratified according to socio-economic levels as good, middle, and low according to local health authorities. Of the 21 PHCs that were providing health care services in the research area, seven were recruited for the study using the simple random sampling technique. Seven PHCs were stratified according to socio-economic status and included in the study, with three PHCs classed as “low,” three PHCs as “middle,” and one PHC as “good.” Of the 68 health clinic units connected to the seven PHCs, 34 were chosen by selecting half of the total number of Community Health Centers (CHCs) affiliated with each PHC region. In the study, 13–15 households from each CHC were visited, and data were collected *via* face-to-face interviews.

In 2017, 30 of the 71 FHCs that provided healthcare services in the region of the previous study in 2004 were included in this study. Of the 30 FHCs stratified according to socioeconomic status, nine FHCs were “good,” seven FHCs were “middle,” and 12 FHCs were “low.” In the study, 26–29 households were visited in each FHC unit and data were collected *via* face-to-face interviews. In determining the sample size of the study, the prevalence of healthcare service use (49% for 2004 and 35% for 2017) and the average number of individuals aged 15 and over [2.89 (≈3)] were calculated for each household for the measurement of general health perception.

In 2004, the size of the sample was based on the rate of healthcare service use, which was accepted as 49% throughout Turkey, and the number of people to be included in the sampling was calculated as 1,288, with an interval of confidence of 95%, α = 0.05, β = 0.20 and effect size of *d* = 0.08, using the NCSS (Statistical and Power Analysis Software-PASS). The number of PHCs in the center of the province (168,064) was compared to the urban population (648,845) to determine the number of people aged 15 and over in each dwelling. It was calculated that there could be ~2.89 (≈3) persons aged 15 and over in each dwelling. Based on this result, it was considered sufficient to include 430 dwellings in the study to achieve a sample size of 1,288 people. In the study, 1,304 people aged 15 and over in 501 households were reached. A questionnaire was provided to each of the 4.03 ± 1.03 people in the household.

**Figure 2:**
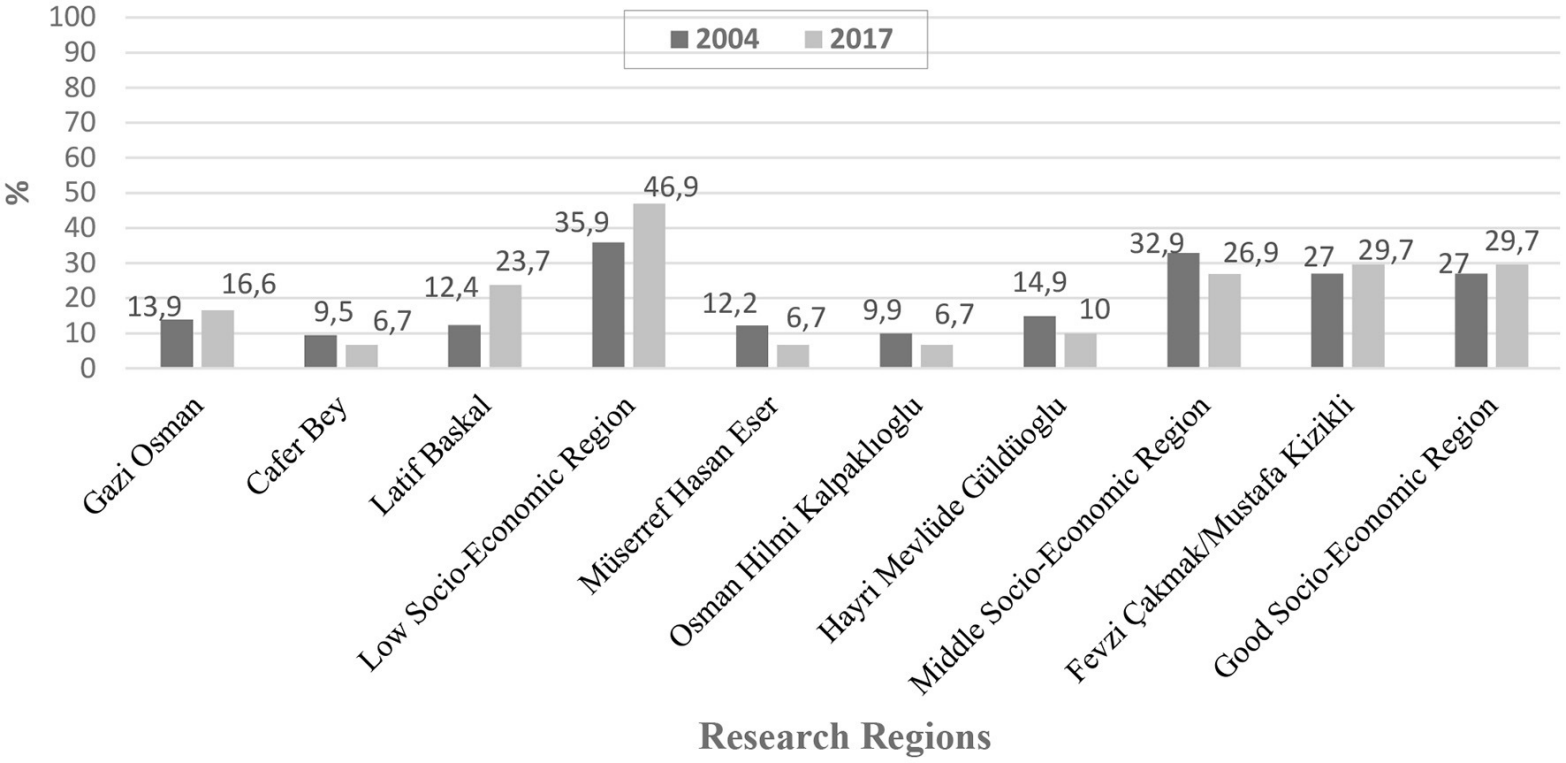
Distribution of the number of people reached in 2004 and 2017.

In 2017, the sample size was determined as 2,000 people; to achieve a minimum of 80% power of representation using the NCSS, the rate of PHC use was accepted as 35%, with a confidence interval of 95%, α = 0.05, β = 0.20 and effect size of d = 0.10. In 2017, it was considered appropriate to include 670 households in the scope of the research to reach the target sample size of 2,000 people, depending on the target of reaching ~3 people in each household. In the study, 1,533 people, aged 15 and over in 801 households were reached. A questionnaire was provided to 3.19 ± 0.98 persons per household (Figure 2).

### Data collection tools

Research data were collected *via* face-to-face interviews, upon visiting the members of the households, through demographic data forms for families and adults (≥15 years) and the elderly (≥65 years), the single-item Self-Rated Health (SRH) question, and the Nottingham Health Profile.

**Self-rated health (SRH)** is measured with the single-item question, “*In general, how would you rate your overall health?*”. The responses were based on a five-point scale, ranging from excellent to poor. For the analyses, where it was considered a continuous variable, “poor” was coded as 1, “fair” as 2, “good” as 3, “very good” as 4, and “excellent” as 5. Regression analyses dichotomized these responses into “good self-rated health” (i.e., excellent, very good, and good) and “poor self-rated health” (i.e., fair and very poor) (1, 2).

**The Nottingham Health Profile (NHP)** is a generic and simple scale designed to measure subjective health status (or QoL) in physical, social, and emotional domains (8). The NHP is composed of two parts. In the first part, there are 38 dichotomous items (yes/no answers) covering six health dimensions: energy (three items), pain (eight items), emotional reactions (nine items), sleep (five items), social isolation (five items), physical mobility (eight items). “No” answers to each statement in the profile are coded as 0 and “yes” answers are coded as 1. Total score ranges for the NHP are from 0 to 600.

In this study, firstly, the “yes” answers given to the scale questions were scored using weighted values, and the possible range of scores for each dimension is 0 to 100 points. In part 1, the scores close to 100 points indicated “poor” perceived health, and those close to 0 points indicated “good” perceived health. In 2004 and 2017, Cronbach's alpha coefficient for the total scale was 0.91 and 0.92, respectively. The six dimensions ranged from 0.71 to 0.87 in 2004 and from 0.72 to 0.89 in the current study, confirming its validity and reliability for the Turkish version.

### Statistical analysis

Data analysis was performed with the statistical package program IBM Corp., 2017. IBM SPSS Statistics for Windows, Version 25.0. Armonk, NY: IBM Corp. The Shapiro-Wilk Test was used to determine the convenience of quantitative variables in a normal distribution. A brief representation of the quantitative variables according to the normal distribution was indicated as the mean, standard deviation, and median (Q1–Q3) of the non-matching variables. The Mann-Whitney U Test was used in the comparison of the two independent groups. The Kruskal Wallis Test was used to compare more than two groups. The Bonferroni Test was used to identify groups that cause differences. Single and multiple binary logistic regression analyses were used to identify the determinants of perceived health status. The dichotomous SRH (good and poor perception) was evaluated as a dependent variable in the model. Variables that showed a significant relationship in univariate analyses, such as age, gender, marital status, monthly household income, educational status, family type, distance from the home to health institutions, perceived health, presence of chronic disease, and hospitalization were evaluated as independent variables. In two regression models for 2017 and 2004, the odds ratio (OR), 95% confidence interval (CI), and Nagelkerke R squared were calculated for each variable. The Hosmer–Lemeshow goodness-of-fit test was used to determine how well the model fits with the data. Categorical variables were shown as percentages and frequencies. The Pearson Chi-Square Analysis was used to examine the relationship between categorical variables. The statistical significance level was accepted at *p* < 0.05.

## Results

A total of 1,304 and 1,533 questionnaires were analyzed in 2004 and 2017, respectively. The mean age was 37.05 ± 15.46 and 39.24 ± 14.51 in 2004 and 2017, respectively. The sample groups in 2004 and 2017 had no statistically significant differences regarding age group or gender (Table 1).

**Table 1:** Distribution of gender and age groups of the respondents in 2004 and 2017.

**Sociodemographic variables**		**2004**		**2017**		**Statistical assessment**	
		***n***	%^*^	***n***	%^*^	**χ^2^**	***p***
Gender	Male	588	45.1	677	44.2	0.229	0.632
	Female	716	54.9	856	88.8		
Age groups	15–44	921	70.6	1,077	70.3	0.303	0.860
	45–64	288	22.1	336	21.9		
	≥65	95	7.3	120	7.8		
Total	1,304	100.0	1,533	100.0

The number of people who responded to the SRH question was 2826. In 2004 and 2017, respectively, the percentage of people who rated their health as excellent was 2.1% vs. 1.8%, very good was 14.6% vs. 14.7%, good was 39.2% vs. 53.5%, fair was 34.9% vs. 25.5%, and very poor was only 9.1% vs. 4.5%. The rate of good health perception increased from 56% in 2004 to 70% in 2017 (*p* < 0.001). In addition, it was found that some sociodemographic and clinical variables were significantly associated with SRH (Table 2). The prevalence of poor SRH was significantly higher in females, those aged 65 and over, illiterates and those who completed primary education only, low-income earners, those with chronic diseases, and those who had used healthcare services or been hospitalized within the 12 months preceding the survey in both 2004 and 2017 (Table 2).

**Table 2:** Patient-reported sociodemographics and clinical outcomes in people aged 15 years and over by their self-rated health (SRH) at baseline.

**Sociodemographic and clinical variables**	**Self-rated health**					
	**2004 (** ***n*** **: 1,304)**			**2017 (** ***n*** **: 1,522)**		
	**Good**	**Poor**	*χ^2^* **/** ***p***	**Good**	**Poor**	*χ^2^**/*****p****
	***n*** **(%)**	***n*** **(%)**		***n*** **(%)**	***n*** **(%)**	
All group	730 (56.0)	574 (44.0)	59.918/ <0.001	1066 (70.0)	456 (30.0)	59.918/ <0.001
**Gender**						
Male	379 (64.5)	205 (35.5)	31.207/ <0.001	506 (75.5)	166 (24.7)	15.854/ <0.001
Female	351 (49.9)	365 (51.0)		560 (65.9)	290 (34.1)
**Age (years)**						
15–24	227 (69.4)	100 (30.6)	57.358/ <0.001	192 (81.0)	45 (19.0)	98.519/ <0.001
25–44	341 (57.4)	253 (42.6)		641 (76.3)	199 (23.7)
45–64	130 (45.1)	158 (54.9)		183 (54.5)	153 (45.5)
≥65	32 (33.7)	63 (66.3)		50 (45.9)	59 (54.1)
**Marital status**						
Single	220 (72.6)	83 (22.4)	53.909/ <0.001	211 (76.2)	66 (23.8)	21.809/ <0.001
Married	475 (52.5)	429 (47.5)		811 (70.1)	346 (29.9)
Divorced/widowed	35 (36.1)	62 (63.9)		44 (50.0)	44 (50.0)
**Education level**						
Illiterate, primary education	305 (44.8)	376 (55.2)	72.488/ <0.001	254 (53.1)	224 (46.9)	94.862/ <0.001
Secondary and high school and faculty	425 (68.2)	198 (31.8)		812 (77.8)	232 (22.2)
**Household monthly income**						
Low	360 (50.6)	351 (49.4)	24.413/ <0.001	64 (55.7)	51 (44.3)	14.995/0.001
Middle	328 (61.2)	208 (38.8)		882 (70.5)	369 (29.5)
Favorable	42 (73.7)	15 (26.3)		120 (76.9)	36 (23.1)
**Presence of chronic diseases**						
No	633 (65.7)	331 (34.3)	140.655/ <0.001	905 (77.7)	260 (22.3)	46.316/ <0.001
Yes	97 (28.5)	243 (71.5)		163 (43.1)	196 (56.9)
**Number of chronic diseases**						
1	88 (31.1)	195 (68.9)	5.166/0.024	138 (50.9)	133 (49.1)	13.583/ <0.001
Comorbidity (2–4)	9 (16.1)	47 (83.9)		25 (28.4)	63 (71.6)
**Use of healthcare services in the last year**						
No	209 (69.9)	90 (30.1)	30.497/ <0.001	209 (82.0)	46 (18.0)	20.746/ <0.001
Yes	521 (51.8)	484 (48.2)		857 (67.6)	410 (32.4)
**Hospitalization in the last year**						
No	669 (58.0)	485 (42.0)	16.133/ <0.001	1,013 (72.4)	376 (27.6)	43.316/ <0.001
Yes	61 (40.7)	89 (59.3)		53 (43.1)	70 (56.9)

### Distribution of HRQoL

The general health perception of people aged 15 years and over was evaluated using the NHP. In 2004, 1,304 people responded to the questionnaire, and 1,508 people responded in 2017. The total NHP score was 30.87 in 2004 and it decreased to 20.34 in 2017. In addition, the NHP total and subdimension scores also decreased to a remarkable level in 2017 compared to 2004. “Energy” and “Physical Mobility” were the highest and lowest scores in 2004, and they decreased from 36.81 to 14.72 (Table 3). Total NHP scores varied significantly by sociodemographic and clinical variables in both 2004 and 2017 (Table 4).

**Table 3:** Nottingham Health Profile Scores of respondents aged 15 and over in 2004 and 2017.

**Nottingham Health Profile dimensions**	**Nottingham Health Profile Scores**				**Statistical assessment**	
	**2004 (** ***n*** **: 1,304)**		**2017 (** ***n*** **: 1,508)**			
	**Mean** ±**SD**	**Median (Q1–Q3)**	**Mean** ±**SD**	**Median (Q1–Q3)**	**Z** ^*^	***P***
Energy	48.89 ± 40.95	39.2 (0–100)	36.81 ± 41.20	24.0 (0–76)	8.016	<0.001
Pain	24.56 ± 32.03	9.9 (0–38.9)	13.58 ± 24.97	0.0 (0–14.8)	10.386	<0.001
Emotional reactions	41.71 ± 32.13	33.3 (11.1–66.6)	24.82 ± 30.12	11.1 (0–44.4)	14.899	<0.001
Sleep	26.98 ± 29.81	22.3 (0–48.9)	19.13 ± 27.56	0.0 (0–34.9)	8.296	<0.001
Social isolation	22.00 ± 28.42	0.0 (0–41.4)	12.98 ± 25.05	0.0 (0–20.1)	10.816	<0.001
Physical mobility	21.07 ± 24.63	10.8 (0–41.3)	14.72 ± 25.09	0.0 (0–21.3)	9.419	<0.001
Total (1st section profile point)	30.87 ± 23.60	26.8 (11.1–48.1)	20.34 ± 22.13	13.8 (0–32.1)	13.408	<0.001

* M-W U, Mann Whitney U Test Z statistics.

Mean ± SD, mean ± standard deviation.

Median (Q1–Q3): (25%−75% percentiles).

**Table 4:** Distribution of total Nottingham Health Profile Scores according to sociodemographic and clinical variables in 2004 and 2017.

**Sociodemographic and clinical variables**	**Total Nottingham Health Profile Score**			
	**2004 (** ***n*** **: 1,304)**		**2017 (** ***n*** **: 1,508)**	
	**Mean** ±**SD**	**Median (Q1–Q3)**	**Mean** ±**SD**	**Median (Q1–Q3)**
**Gender**				
Male	24.29 ± 20.72	19.4 (7.4–36)	16.46 ± 20.71	9.6 (0–24.4)
Female	36.27 ± 24.45	34.12 (15.4–55)	23.39 ± 22.74	18 (1.85–37.9)
*M-W U/p*	8.970, <0.001		6.493, <0.001	
**Age groups**				
15–24	24.45 ± 22.13	18.7 (6.4–37.7)	20.05 ± 20.11	15.0 (2–34.1)
25–44	29.53 ± 22.45	25.5 (10.8–45.2)	16.35 ± 18.85	10.1 (0–26)
45–64	35.11 ± 24.45	33.4 (13.9–55.2)	25.20 ± 23.77	19.13 (4–37.8)
≥65	48.47 ± 22.20	43.6 (32.1–65.3)	39.19 ± 32.65	39.2 (9.3-62.6)
*K-W H/p*	87.813, <0.001		73.635, <0.001	
**Marital status**				
Single	11.28 ± 20.27	17.2 (5.8–32.4)	21.53 ± 20.71	17.8 (4–34.3)
Married	22.13 ± 24.02	28.1 (12.1–48.6)	19.20 ± 22.00	12.4 (0–29.3)
Divorced/widowed	41.72 ± 27.71	49.3 (29.7–67.9)	31.73 ± 24.99	28.1 (10.8–46)
*K-W H/p*	89.114, <0.001		29.900, <0.001	
**Education level**				
Illiterate	52,72 ± 23.00	53.5 (35.8–71.6)	39.86 ± 28.23	39.5 (13.4–60.4)
Primary education	33.61 ± 22.33	30.4 (15.2–49.9)	26.28 ± 23.17	21.8 (7.8–40.7)
Secondary and high school	24.02 ± 20.66	19.4 (7.4–37.4)	16.69 ± 19.68	10.1 (0–26.4)
University	17,49 ± 17,98	12.8 (2.4–26.6)	13.74 ± 16.38	7.43 (0–22.9)
*K-W H/p*	218.373, <0.001		136.628 <0.001	
**Household monthly income**				
Lower	34.23 ± 23.82	30.1 (14.8–52.5)	28.10 ± 28.79	19.5 (6.5–39.3)
Middle	27.61 ± 22.68	22.4 (7.9–43.2)	20.41 ± 21.84	13.8 (0–33.2)
Good	18.50 ± 21.06	8.4 (2–33.3)	14.17 ± 16.39	9.5 (0–23.8)
*K-W H/p*	45.961, <0.001		18.052, <0.001	
**Presence of chronic disease**				
No	25.95 ± 21.47	45.9 (25.8–63.4)	16.79 ± 19.86	26.7 (12.1–50.1)
Yes	44.81 ± 23.82	21.1 (7.9–39.1)	32.14 ± 25.04	10.1 (0–26.1)
*M-W U/p*	12.277, <0.001		11.089, <0.001	
**Use of healthcare services the last year**				
No	13.87 ± 20.37	19.8 (9.8–35.6)	12.47 ± 18.81	2 (0–19.4)
Yes	32.82 ± 24.46	28.9 (11.6–51.2)	16.39 ± 26.07	16.3 (1.9–34.5)
*M-W U/p*	4.903, <0.001		7.559, <0.001	
**Hospitalization in the last year**				
No	29.19 ± 22.87	25.3 (10.1–45.9)	19.08 ± 21.44	12.5 (0–30.0)
Yes	43.75 ± 25.16	42.9 (23.6–64.8)	34.75 ± 24.76	28.3 (17.5–52.2)
*M-W U/p*	6.677, <0.001		7.366, <0.001	

M-W U, Mann Whitney U Test Z statistics; K-W H, Kruskal Wallis-H Test F statistics.

Mean ± SD, mean ± standard deviation.

Median (Q1–Q3): (25%−75% Percentiles).

### SRH and its relation to HRQoL

SRH was closely associated with HRQoL in both 2004 and 2017. The NHP total and subdimension scores were significantly different in individuals with positive self-perceived health status when compared to people with negative self-perceived health status. Self-perceived health was more prominent in all dimension scores for those who performed well. Likewise, while the NHP total score was 20.22 in those with good general health perception in 2004, this decreased to 13.91 in 2017 (*p* < 0.001). In 2017, the levels of QoL related to pain, social isolation, and physical mobility were highest in subjects with good SRH (Table 5).

**Table 5:** Nottingham Health Profile Scores in patients aged 15 and over according to their self-rated health (SRH) at baseline.

**NHP dimensions**	**Self-rated health**					
	**2004 (** ***n*** **: 1,304)**			**2017 (** ***n*** **: 1,508)**		
	**Good**	**Poor**	**Z** ^*^ **/** ***p***	**Good**	**Poor**	**Z** ^*^ **/** ***p***
	***x*** ±**SD**	***x*** ±**SD**		***x*** ±**SD**	***x*** ±**SD**	
Energy	32.88 ± 36.02	69.24 ± 37.70	15.994, <0.001	27.12 ± 36.91	60.19 ± 41.65	14.004, <0.001
Pain	11.16 ± 21.10	41.60 ± 35.32	17.749, <0.001	7.51 ± 17.39	28.20 ± 33.11	15.040, <0.001
Emotional reactions	41.71 ± 32.13	30.70 ± 28.68	13.911, <0.001	18.48 ± 25.52	40.10 ± 34.56	12.060, <0.001
Sleep	18.47 ± 24.84	37.79 ± 32.05	11.291, <0.001	13.59 ± 23.34	32.48 ± 32.02	11.792, <0.001
Social isolation	16.74 ± 25.37	28.70 ± 30.63	7.784, <0.001	8.54 ± 19.91	23.69 ± 31.98	10.469, <0.001
Physical mobility	11.37 ± 18.35	33.39 ± 26.05	16.427, <0.001	8.32 ± 18.51	30.31 ± 31.51	15.684, <0.001
Total (1st section profile point)	20.22 ± 18.11	44.41 ± 22.83	18.368, <0.001	13.91 ± 16.90	35.84 ± 25.33	16.627, <0.001
Overall POINT	30.87 ± 23.60		47.231, <0.001	20.34 ± 22.13		35.693, <0.001

* Mann Whitney U Test, Z statistics.

x ± SD: mean ± standard deviation.

### Determinants with SRH

The common determinants that increased negative health perception in 2004 and 2017 were being female (1.4–1.5 times higher), having at least one chronic disease (3.4–2.7 times higher), and having completed primary education only (2.7–2.8 times higher). Whereas being married (1.7 times higher), use of healthcare services in the last year (1.8 times higher), and middle income (2.3 times higher) were variables specific to 2004, being between the ages of 45 and 64 years (2.3 times higher) and hospitalization in the last year (2.4 times higher) were the main factors associated with poor health perception specific to 2017. However, living 500–1,000 m from the nearest health institution was the main protective factor (1.5–1.7 times higher) against poor health perception in both 2004 and 2017 (Tables 6, 7).

**Table 6:** Univariate and multiple logistic regression (Backward-Wald Method) analyses for models predicting poor self-rated health in 2004 (*n* = 1,304).

**Predictor variables**	**Univariate**				**Multivariate**			
	**Wald**	**OR** ^*^	**95% CI** ^**^	**Sig**.	**Wald**	**OR** ^*^	**95% CI** ^**^	**Sig**.
**Gender**								
Male		1				1		
Female	30.922	1.886	1.508–2.358	<0.001	7.207	1.414	1.098–1.820	<0.001
**Chronic disease**								
Absent		1				1		
Present	129.014	4.791	3.656–6.278	<0.001	69.581	3.456	2.582–4.624	<0.001
**Hospitalization**								
Absent		1			**-**	**-**	**-**	**-**
Present	15.685	2.013	1.424–2.845	<0.001	NS	NS	NS	NS
**Age groups**								
15–24		1			**-**	**-**	**-**	**-**
25–44	12.763	1.684	1.265–2.242	<0.001	NS	NS	NS	NS
45–64	36.230	2.759	1.983–3.839	<0.001	NS	NS	NS	NS
≥65	36.431	4.469	2.748–7.267	<0.001	NS	NS	NS	NS
**Marital status**								
Single		1				1		
Married	36.234	2.394	1.802–3.181	<0.001	12.390	1.762	1.285–2.416	<0.001
Divorced/widowed	39.023	4.695	2.890–7.628	<0.001	3.726	1.722	0.992–2.991	0.054
**Education level**								
Illiterate		1				1		
Primary school	21.805	0.427	0.298–0.610	<0.001	11.237	2.729	1.517–4.909	0.001
Secondary school	53.375	0.230	0.155–0.341	<0.001	5.910	1.809	1.122–2.916	0.015
High school and University	48.464	0.158	0.094–0.265	<0.001	1.883	1.419	0.861–2.340	0.170
**Proximity to health facility (meters)**								
>1,000 m^*^		1				1		
<500 m	0.005	0.990	0.750–1.307	0.944	0.927	0.860	0.632–1.169	0.336
500–1,000 m	8.323	0.686	0.531–0.886	0.004	9.610	0.639	0.481–0.848	0.002
**Family income**								
Low		1				1		
Middle	13.723	0.650	0.518–0.817	<0.001	5.425	2.258	1.138–4.483	0.020
Favorable	10.495	0.366	0.200–0.673	<0.001	1.484	1.526	0.773–3.014	0.223
**Family type**								
Traditional		1			**–**	**–**	**–**	**–**
Nuclear	0.692	0.885	0.655–1.179	0.405	NS	NS	NS	NS
Shattered	1.742	1.395	0.851–2.285	0.187	NS	NS	NS	NS
**Use of healthcare services in the last year**								
Absent		1				1		
Present	29.734	2.157	1.636–2.844	<0.001	14.331	1.798	1.327–2.436	<0.001

Proximity to health facility ref: >1,000 m.

* OR, odds ratio;

** 95% CI, confidence interval; NS, not significant; Age, family type, and hospitalization variables, which showed a significant relation in univariate regression but they were not evaluated in multiple regression because of not showing permanent relation.

**Table 7:** Univariate and multiple logistic regression (Backward-Wald method) analyses for models predicting poor self-rated health in 2017 (*n* = 1,508).

**Predictor variables**	**Univariate**				**Multivariate**			

	**Wald**	**OR** ^*^	**95% CI** ^**^	**Sig**.	**Wald**	**OR** ^*^	**95% CI** ^**^	**Sig**.
**Gender**								
Male		1				1		
Female	15.948	1.592	1.267–2.000	<0.001	11.903	1.568	1.214–2.024	0.001
**Chronic diseases**								
Absent		1				1		
Present	122.665	4.165	3.236–5.362	<0.001	50.822	2.797	2.108–3.711	<0.001
**Hospitalization**								
Absent		1				1		
Present	43.708	3.590	2.458–5.245	<0.001	17.448	2.461	1.613–3.755	<0.001
**Age groups**								
15–24		1				1		
25–44	41.019	1.325	0.923–1.901	<0.001	1.398	1.256	0.861–1.831	0.237
45–64	28.331	3.567	2.417–5.264	<0.001	15.104	2.340	1.524–3.592	<0.001
≥65	76.736	4.089	2.434–6.868	<0.001	0.691	1.326	0.682–2.577	0.406
**Marital status**								
Single		1			–	–	–	–
Married	3.335	1.328	0.979–1.800	0.068	NS	NS	NS	NS
Divorced/widowed	17.782	2.979	1.794–4.948	<0.001	NS	NS	NS	NS
**Education level**								
Illiterate		1				1		
Primary school	39.894	0.278	0.187–0.414	<0.001	11.126	2.806	1.530–5.144	0.001
Secondary school	65.941	0.171	0.112–0.262	<0.001	0.602	1.167	0.790–1.723	0.438
High school and university	49.654	0.172	0.106–0.281	<0.001	0.150	0.921	0.608–1.396	0.699
**Proximity to health facility (meters)**								
>1,000 m^*^		1				1		
<500 m	0.646	1.126	0.843–1.504	0.422	1.307	1.200	0.878–1.642	0.253
500–1,000 m	13.199	0.607	0.463–0.794	<0.001	14.820	0.561	0.418–0.753	<0.001
**Family income**								
Low		1			–	–	–	–
Middle	9.919	0.533	0.360–0.788	0.002	NS	NS	NS	NS
Favorable	12.770	0.381	0.224–0.647	<0.001	NS	NS	NS	NS
**Family type**								
Traditional		1			–	–	–	–
Nuclear	9.297	0.650	0.493–0.857	0.002	NS	NS	NS	NS
Shattered	0.209	0.880	0.508–1.524	0.648	NS	NS	NS	NS
**Use of healthcare services in the last year**								
Absent		1			–	–	–	–
Present	19.371	2.162	1.534–3.048	<0.001	NS	NS	NS	NS

Proximity to health facility ref: >1,000 m.

* OR, odds ratio;

** 95% CI, confidence interval.

NS, not significant; Marital status, family income, family type, and use of health services variables, which showed a significant relation in univariate regression but they were not evaluated in multiple regression because of not showing permanent relation.

In the 2004 study, hospitalization (2.0 times higher) and age (1.6–4.5 times higher) significantly increased poor health perception in the single regression analysis and were dropped from the model because a significant relationship could not be maintained in the multiple regression analysis. In the 2017 study, in univariate regression analysis, use of healthcare services, which increased poor health perception by 2.2 times, and being separated from a spouse, which increased it by 2.9 times, were dropped from the model because they could not maintain a significant relationship in the multiple regression analysis. The variables of middle- and good-income levels (0.533 and 0.381) and nuclear family structure (0.650), which provided protective effects against poor health perception, did not show a significant relationship in multiple regression and therefore could not persist in the model.

## Discussion

To the best of our knowledge, this study is one of the limited number of studies in which both parameters, SRH and HRQoL, are used together to determine the general health perception in the general population, and in this context, the factors affecting both are defined. Furthermore, it presents a time-dependent change in the study with results that define the factors affecting perceived individual health using the same measurement tools in the same region.

The findings of this study show that the prevalence of good SRH increased significantly over time. In fact, the rate of respondents who had “good” health perception, which was 56.0% in 2004, increased to 70.0% in 2017 (Table 2). In Turkey, according to the 2019 OECD health statistics, 68.8% of the population rated their health as good. In this context, it can be assumed that the rate of good health perception obtained from this study is comparable with the overall rate reported for Turkey. The rate of good SRH obtained in both of the current studies in Turkey is higher than Korea, Japan, Portugal, and Poland and is almost homogeneous with other OECD countries (Austria, Finland, Denmark, and Luxemburg), but it is lower than New Zealand, the USA, Switzerland, Norway, Spain, and Australia (26). The differences might be partially due to the methodologies used for measuring SRH and reported SRH status being exposed to biological, psychological, and social dimensions, such as age, sex, place of residence, education, occupation, level of income, and lifestyle factors, as well as the possibility of being affected by perceptual differences and cultural factors (1, 2, 27). In addition, previous studies conducted by Dong et al. (28) indicate that good SRH is higher in married, non-smoking, and non-alcohol users. A study carried out by Liu et al. (12) reports that marriage is the main determinant of good SRH. Similarly, Darviri et al. (29) revealed that a healthier diet and regular exercise are closely related to good health perception. In contrast to these studies, Orea et al. (13) reported that strong physical activity and adequate nutrition are among the determinants of poor health perception. Coinciding with the studies in the literature (12, 30–33), the present study reveals that the prevalence of poor SRH is significantly higher in females, those who are of advanced age, those with low income and a low level of education, those with chronic diseases, and those who had used healthcare services or been hospitalized within the 12 months prior to the survey in both 2004 and 2017 (Table 2).

### SRH and HRQoL

In this study, self-perceived overall health status was evaluated using the NHP. It was observed that the total and subdimension scores, obtained from the profile in 2017, decreased significantly compared to 2004. This confirms that people who rated their health had experienced a positive change in all domains (Table 3).

However, it was observed that self-perception of overall health is closely related to QoL. Better HRQoL was found to be consistent with better SRH status. Similarly, as the NHP total and subdimension scores improve, positive self-perceptions of health increase significantly. In particular, pain, social isolation, and physical mobility QoL scores are significantly better in those who rate their health positively. On the other hand, energy, emotional reactions, and sleep QoL scores were found to be better in 2017 compared to 2004 in individuals with a good perception of their health, but the improvement in scores is relatively low when compared to other areas (Table 5). This is consistent with the findings of a study conducted by Uutela et al. (34) and Kara (35) on patients with chronic diseases, which found that NHP dimensions for pain, energy, emotional reactions, and mobility were significantly associated with health perception. Previous studies have found that, similar to our study findings, dynamism and daily activities are important components of QoL and that mental and physical functions, physical exercise, and rich social relationship networks are positively correlated with QoL levels (28, 36, 37).

In this study, the relationship between HRQoL and self-reported health status may be mediated by several factors in both periods (Tables 2, 4), which has been confirmed in other studies (35, 38–43). In this regard, our study reveals that sociodemographic and clinical factors not only mediate the change in NHP scores but also impact the deterioration of SRH perception. The findings, in relation to impaired HRQoL and poor perception of health, are significantly associated with females, the elderly, widowed and divorced people, those with a low income and level of education, those with one or more chronic diseases, and those who had been hospitalized within the 12 months prior to the survey (Tables 2, 4). It is known that men and women typically have different health outcomes when exposed to similar risks, which may account for the gender disparity in reporting poor SRH and impaired QoL. High educational attainment often explains the beneficial relationship between education and health directly through the improvement of health due to rewarding employment, favorable social and economic circumstances, and the adoption of healthy lifestyle habits. Respondents with higher levels of education are more aware of health issues and the importance of maintaining their protective actions against poor health perception and reduced quality of life. Poor perception of health and impaired QoL in widowed or divorced individuals may be associated with a lack of emotional and practical support and a feeling of loneliness. However, marriage might be considered a protective factor against these deprivations. When sociodemographic characteristics are used as control variables in people with a poor perception of their general health, we can conclude that the severity of fatigue, inadequate social participation, physical activity limitations, sleep dissatisfaction, and emotional reaction problems are significantly higher in the above-mentioned sensitive groups. Consistent with our results in previous studies, physical activity, social participation, and sleep quality have been defined as the main determinants affecting both QoL and SRH status (29, 40). In some studies (16, 44), it has been found that sleep dissatisfaction is closely related to poor SRH and impaired QoL. However, in other studies (40, 41), it is emphasized that physical activity levels and social participation may improve perceptions of SRH and QoL in support of the above-mentioned findings.

### Comparison of predictors of poor SRH

In this study, the determinants of poor SRH were evaluated using single and multiple regression analysis (Tables 6, 7). The regression analysis revealed that the rate of poor health perception is higher in women, those with a low level of education, and those with chronic disease, supporting the univariate relationship results. The risk of negative health perception due to being female has increased over time; while the relative risk was 1.4 times higher in 2004, it was found to be 1.8 times higher in 2017. In accordance with the findings in our study, some research has consistently shown that gender has a significant influence on poor SRH status (28, 30, 45, 46). These studies state that poor SRH is between 1.2 and 3.4 times higher in females when compared to their male peers. Our study findings may have been affected by the fact that most of the women in the study group did not work in a job that generates an income (65% housewives), had a low level of education (62.2% illiterate and individuals who completed primary education only), and were of advanced age (53.4% aged 65 and over). These results indicate that more attention is needed on women's health and appropriate public health interventions should be implemented to improve their health and social status in Turkey.

Regression analysis revealed a significant interaction between poor SRH and literacy in this study. The relative risk of negative health perception due to primary education level increased at a similar rate over time. The odds ratio for poor SRH was 2.8 times higher in 2017 and 2.7 times higher in 2004. In consistency with our findings, Stanojevic Jerkovic et al. (47) reported that the completion of primary education only is the strongest factor [OR: 4.3 (2.5–7.3)] associated with poor SRH. However, some studies indirectly support our findings; Dong et al. (28) stated that higher education is a protective factor (OR: 0.9 vs. 0.7) against poor SRH. Orea et al. (13) reported that the relative risk of poor health perception was 0.70 (0.5–0.8) for university graduates in comparison with 0.75 (0.6–0.9) in those who completed secondary education (Tables 6, 7).

In this study, while the odds ratio for poor SRH was 3.4 times higher in 2004 in those who had one or more chronic diseases, it decreased to 2.8 times in 2017 (Tables 6, 7). Previous studies have indicated that the relative risk of poor perception of health is higher, between 1.3 and 2.6 times, in people who had one or more chronic diseases. In patients with chronic diseases, the odds ratio for poor SRH was found to be 1.3–1.4 times higher by Orea et al. (13), 2.6 times higher by Stanojevic Jerkovic et al. (47), 2.0 times higher by Liu et al. (12), 2.3 times by Wang et al. (45), and 1.6 times higher by Cau et al. (46). These studies correspond with the current study's findings, which show that subjective health perception also depends on objective health (3, 48).

In this study, regression analysis revealed that place of residence is a significant determinant of self-perceived overall health status. Living near a health center (500–1,000 m) is conducive to better (OR: 0.63 vs. 0.56) health perception (Tables 6, 7).

Consistent with this result, previous studies (45, 49) have demonstrated that increased physical access to healthcare services also influences respondents' reported health.

Negative health perception, which was found to be 1.7–4.5 times higher in all age groups in single regression in 2004, was omitted from the model because it did not show a significant relationship in the multiple regression step. However, the perception of poor health, which was seen at 1.3–4.0 times higher in all age groups in single regression in 2017, was only found to be 2.3 times higher in the 45–64 age group in the multiple regression analysis (Tables 6, 7). In previous studies, Dong et al. (28) found that in those aged 75 and older, the odds of reporting poor health were 4.9 times higher than in those aged 18–24 years old. Liu et al. (12) reported that poor SRH was 1.9 times higher in people aged 41–56 and 3.0 times higher in those aged 57–72. Wang et al. (45) showed that poor perceived health was 1.8 times higher in people aged 45, while it was 3.9 times higher in those aged 65.

The increase in negative health perception due to hospitalizations in the year prior to the study showed a significant relationship only in univariate regression analysis in 2004. In contrast, an increase in negative health perception (2.07-fold) due to hospitalizations was found to be a significant relationship in both single and multiple regression analysis in the 2017 study (Tables 6, 7). Previous studies (30, 45) have consistently demonstrated that hospitalization is associated with poorer SRH status, which is consistent with our findings. In these studies, it is reported that the odds ratio for the poor perception of health is 2.2 times and 1.9 times higher in those who had been hospitalized than in those not hospitalized in the year prior to the study, respectively.

In the 2004 study, multivariate regression analysis revealed that the strongest factors associated with poor SRH were middle household income (OR: 2.2, 1.1–4.4), being married (OR: 1.7, 1.2–2.4), and using healthcare services in the 12 months prior to the survey (OR: 1.8, 1.3–2.4) (Table 6). In the single regression analysis conducted in the 2017 study, while the determinants of the increase in poor health perception included middle and favorable income levels (0.53- and 0.58-fold), being divorced or widowed (2.9-fold), and use of healthcare services in the 12 months prior to the survey (2.2-fold), the variables mentioned above were dropped from the model because the significant relationship did not persist in the multiple regression analysis (Table 7).

In the literature, some studies indicate that marital status plays a decisive role in poor SRH, which is consistent with our previous study findings. In this context, the components of marital status that affect negative health perceptions differ from study to study. For example, Liu et al. (12) showed marriage to be a protective (OR: 0.8, 0.7–0.9) factor against negative health perception, and Cau et al. (46) revealed that poor health perception was 4.7 times higher in single people and 2.1–1.8 times higher in widowed or divorced people. However, Khabir et al. (33) reported that the ratio of poor health perception was 1.8 (1.6, 2.0) times higher in married people and 4.0 (3.3, 4.9) times higher in widowed or divorced people.

In addition, similar to the findings of our 2004 study, previous studies (50–52) have consistently revealed that the use of healthcare services is associated with poorer SRH status. These studies state that people who perceive their own health status as poor are more likely to use healthcare services, 76.9 times (50) and 3.8 times (51) more than those who perceive their health status as good in the 12 months prior to the survey. In other words, poor SRH status has also been shown to be independently predictive of higher healthcare utilization rates (53).

As a result, it's possible that the variables covered by the health transformation program, which has been in effect in the study's region of Turkey since 2003, are strongly related to the gradual improvement in self-reported health and quality of life, indirectly. Furthermore, in our study, the increase in the use of healthcare services from 79.6% in 2004 to 84.8% in 2017 (*p* < 0.001) and the increase in the use of primary healthcare centers from 30.3% to 45.8% (*p* < 0.001) can be attributed to the relative effect of the implementation of the health transformation program in the research region. It is thought that the improvement in positive health perception and quality of life may have been relatively affected by the changes in the demographic and economic characteristics of the participants over time as well as the increased physical and financial access to primary healthcare services.

## Conclusions

Based on the results of this study, the levels of good self-rated health have significantly improved over time. In the same time period, the mean total NHP score decreased from 30.87 (±23.60) to 20.34 (±22.13). The improvement in the total NHP and subdimension scores support an increase in good health perception. Poor SRH is associated with being female, being 45–64 years old, having a low level of education, having chronic diseases, and having been hospitalized. Proximity to health facilities is the main protective factor against poor SRH. According to the findings of the study, local and national governments can be informed about the factors that influence negative health perception and take steps to improve physical, psychosocial, and economic health in disadvantaged groups. Thus, preventive measures can be taken in order to establish health-promoting policies and improve public health.

## Data availability statement

The original contributions presented in the study are included in the article/supplementary material, further inquiries can be directed to the corresponding author.

## Ethics statement

This study was approved by the Ethics Committee in Clinical Research of Human Subjects at Erciyes University, Faculty of Medicine (Decision date and no: 2005/240 vs. 2015/399) and permission was obtained from the governor's office of Kayseri. All respondents provided written consent to participate in both studies in 2004 and 2017 before data collection. The patients/participants provided their written informed consent to participate in this study.

## Author contributions

VS: idea, concept, design, supervision/consulting, data collection, processing analysis, interpretation, and paper writing. MN: literature review. FC: idea, concept, design, interpretation, and supervision. FE: processing analysis and interpretation. All authors contributed to the article and approved the submitted version.
